# Indigenous and traditional plants: South African parents’ knowledge, perceptions and uses and their children’s sensory acceptance

**DOI:** 10.1186/1746-4269-9-78

**Published:** 2013-11-25

**Authors:** Marinka van der Hoeven, Jennifer Osei, Minrie Greeff, Annamarie Kruger, Mieke Faber, Cornelius M Smuts

**Affiliations:** 1 Africa Unit for Transdisciplinary Health Research (AUTHeR); Faculty of Health Sciences, North-West University, Potchefstroom Campus, Private Bag ×6001, Potchefstroom 2520, South Africa; 2 Centre of Excellence for Nutrition (CEN); Faculty of Health Sciences, North-West University, Potchefstroom Campus, Private Bag ×6001, Potchefstroom 2520, South Africa; 3 Nutritional Intervention Research Unit, Medical Research Council (MRC), PO Box 19070, Tygerberg 7505, South Africa

**Keywords:** Indigenous plants, Traditional plants, African leafy vegetables, Knowledge, Perception, Use, Sensory evaluation, Parents, Primary school children, South Africa

## Abstract

**Background:**

The dietary shift from indigenous and traditional plants (ITPs) to cash crops and exotic plant food sources increases the risk of malnutrition and other nutrition-related non-communicable diseases, especially in poor rural communities. Farm communities in South Africa have been associated with poor nutritional status and extreme poverty. ITPs have been found to be affordable sources of several micronutrients. However, knowledge of and the use of these plants are declining, and little is known about the child’s acceptance of dishes prepared with ITPs. This knowledge can be used to improve the general acceptance of ITPs. This study aimed to gain insight into parents’ knowledge and perceptions and their use of ITPs in a farming community in the North West Province and to assess children’s acceptance of and preference for dishes made with African leafy vegetables (ALVs) and Swiss chard.

**Methods:**

Parents (n = 29) responsible for food preparation for children in grade 2 to 4 in two schools were purposively selected for four focus group discussions. A sensory evaluation assessed the children’s (n = 98) acceptance of, preference for and intended consumption of dishes made with leafy vegetables. The dishes were made of *Amaranthus spp*., *Cleome gynandra*, *Cucurbita maxima*, *Vigna unguiculata* and *Beta vulgaris*.

**Results:**

Parents mentioned 30 edible ITPs during the focus group discussions. Parents had knowledge of available ITPs and their use as food. Location, seasonal variation and rainfall affected the availability of and access to ITPs. Sun-dried ITPs were stored in sacks for later use. ITPs were perceived as healthy, affordable and delicious, hence acceptable to the parents. The children also evaluated the dishes made with ALVs as acceptable in terms of colour, smell and taste. Swiss chard was preferred, most likely because of the children’s exposure to this vegetable. Children indicated that they would like to eat these leafy vegetables twice a week.

**Conclusion:**

These results look promising for the promotion of ITPs as a strategy to reduce malnutrition in rural farm communities and for potential inclusion of these micronutrient-rich ALVs in school feeding programmes to improve the nutritional status of children.

## Background

Over millennia, indigenous and traditional plants (ITPs) have been the main source of food for many rural communities. However, colonial economies and post-independence development schemes placed greater emphasis on the production and consumption of cash crops, introduced foods that led to the displacement of indigenous food crops and caused subsequent changes in the diet of African people [1]. Their food patterns reflected an increasing intake of a limited number of domesticated plant staples, while intake of the edible wild plant species that once sustained health and nutritional status was reduced [2]. It is evident that urbanisation has contributed to a decline in knowledge of the usefulness of ITPs, hence the reduction in the consumption of these foods. This dietary change, especially in poor rural communities, put people at risk of malnutrition and other nutrition-related non-communicable diseases. According to the United Nations Children’s Fund conceptual framework on malnutrition, the underlying causes of malnutrition and death in children are poor household food security, inadequate maternal and child care, insufficient health services and an unhealthy environment or lack of education and information [3]. The South African National Food Consumption survey of 1999 showed that a large number of children had inadequate intake of energy, vitamin A, vitamin C, thiamine, riboflavin, niacin, vitamin B6, vitamin B12, folic acid and zinc [4]. It also showed that rural children were worse off than those who lived in urban areas. Lemke stated that in South Africa, with regard to socio-economic status, health status, household nutrition security and education, farm worker households tended to be most vulnerable among all groups [5]. ITPs can play an important part in alleviating hunger and malnutrition. They are important sources of micronutrients, including vitamins A and C, iron and other nutrients, and are sometimes better nutritional sources than modern vegetables [6]. Modernisation of South African rural communities has led to people perceiving ITPs as inferior. Faber *et al*. reported that African leafy vegetables (ALVs) were often regarded as a poor people’s food in South Africa [7]. Labels such as “backward knowledge” have been linked to traditional vegetables and associated knowledge, thus discouraging the youth from learning about them [8,9].

Knowledge of the use of indigenous plants needs urgent scientific investigation and documentation before it is irreversibly lost to future generations [6]. Several studies in South Africa have reported a decline in the use of ITPs [10-12]. However, Shackleton reported the frequent use of wild edible herbs among rural communities [13]. Faber *et al*. [7] concluded that availability and access to nutrition-related uses of ALVs are content-specific, with inter- and intra-provincial rural/urban differences. Different communities may have different perceptions and beliefs about the ITPs that grow in their area and this affects the consumption and use of the plants. Sensory characteristics of food, such as appearance, smell, texture and taste, also play an important role in people’s decision to consume a particular food. Research on the acceptability of food is needed to determine the impact of taste and preference on dietary intake patterns of consumers that can be used to improve the general acceptance of ITP foods [14].

As information collected during small studies within a specific area cannot be generalised to the entire South African population, the objectives of this contextual study were to gain insight into the parent [1] ’s knowledge and perceptions of and their use of ITPs in a farming community in the North West Province and to assess children’s acceptance of and preference for dishes made with ALVs and Swiss chard.

### Methodology

#### Research design

The study was conducted in two phases. The first phase used a qualitative interpretive description approach [15] to explore and describe parents’ knowledge and perceptions and their use of ITPs. Household socio-demographic characteristics were also obtained from these parents. The second phase used a quantitative cross-sectional approach in the form of sensory evaluation to assess children’s acceptance of and preference for dishes made with ALVs and Swiss chard.

### Setting

**Figure 1:**
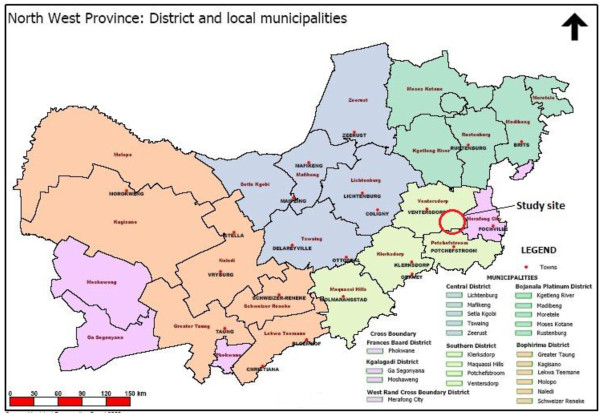
Map indicating the study site in the North West Province of South Africa (adapted from 17).

The North West Province of South Africa is approximately 116 320 square kilometers in area and almost all its rainfall occurs in the summer months between October and April. Average rainfall of 539 mm per annum decreases from east to west. There is a short growing season for frost-sensitive crops between October (last cold) and the end of April (first frost). Regular droughts occur in this province. Sixty percent of the province’s 3.2 million inhabitants live in rural areas [16,17]. According to the North West Province State of the Environment Report in 2002, mining and agriculture, including both crop cultivation and livestock production systems, were the two most important economic sectors. In the same year, the estimated unemployment rate was 38% and approximately a third of the population was illiterate [17]. According to Cloete *et al*., 53% of the population lives in poverty and 41% is economically dependent on social funding from government [18]. The current study was conducted within the infrastructure of two farm primary schools in a rural area approximately 50 kilometres from Potchefstroom in the south–eastern part of the North West Province (Figure 1). The main farming activities in this area include maize, sunflower and chicken farming. Both these primary schools were located in similar farm surroundings (with only very small shops, so-called tuck shops) and were fully sponsored by the South African Department of Education and by the farm owners themselves. One school was situated approximately 25 kilometres from an urban area with markets and grocery stores, the other school approximately 35 kilometres from such an area. Schools were chosen as entry point because they are closely linked to the community.

## Ethical considerations

Ethical approval was granted by the Ethics Committee of the North-West University (NWU-00033-09-A1). Permission to conduct the study was granted by the Department of Education of the North West Province (Dr Kenneth Kaunda district) and the school governing bodies of the two schools. Several parent meetings, in the preferred language of the parents, were held at the school premises to explain the purpose and procedures of the study, and to answer any questions that the parents had. Potential participants were invited to participate in the study and were asked to sign an informed consent form (illiterate people made a cross in front of a witness) agreeing that they themselves and their children would participate in the study. Only children who obtained parental consent and gave assent for the study were included. Potential participating parents were assured of data confidentiality and that the data would be used for the sole purpose of the study. Participation was voluntary and the participants could withdraw at any time without any consequences.

### Phase 1: Parent’s knowledge, perceptions and use of indigenous and traditional plants

#### Sample

Prospective households from which to recruit a purposive voluntary sample of participants were identified through the two primary schools [19]. Two focus group discussions per school were planned, with the possibility of increasing this number if data saturation was not reached. Participants were recruited through house visits. The participant had to be a parent or primary caregiver (hereafter called parent) of a child attending grade 2 to 4 at the selected primary school, responsible for procuring and preparing food in the household, living in the selected community and 18 years or older. After the parent meetings and a week before the focus group meeting, the prospective participant was verbally informed about the research again and invited to participate. If the prospective participant indicated not being available for the focus group, a different parent was asked to participate. Although eight parents were invited to participate per focus group, only one focus group consisted of eight participants. The other three focus groups had seven participants each. Repetition in knowledge and themes was noted after the third focus group discussion. The fourth focus group discussion did not provide any significant new information, therefore no more parents were selected to participate.

#### Data collection

Household socio-demographic characteristics data for the 29 parents participating in the focus group discussions was collected by means of a structured questionnaire prior to the focus group discussions.

The focus group discussions were held in a classroom at the two schools during school holidays. The school environment was chosen in order to make participants feel that they were in an environment to which they were already used. Transport was provided to get to the schools. All participants received refreshments before and after the focus group discussions and were given a small monetary incentive for their participation. Participants were seated in a circle. This allowed them to see one another during the discussions and it also encouraged a sense of group atmosphere and bonding. All focus groups were conducted in the local language (Setswana), audio-recorded and transcribed verbatim. Each focus group was conducted by an interviewer and an assistant moderator, responsible for operating the tape, making observations and taking notes. A photo atlas from the South African Agricultural Research Council with pictures of ITPs [20] was used to assist participants to identify the ITPs and to connect the common names of the plants to their botanical names. All botanical names used in this manuscript have been verified with the International Plant Names Index and the World Checklist of Selected Plant Families [21,22].

The focus groups followed a semi-structured format to ensure accuracy in topics covered across the different groups and still permit a certain level of flexibility within the group [23]. A discussion guide with six open-ended questions was designed to collect data on the knowledge and use of ITPs. The interview guide was discussed with other experts in the field, adjusted and pilot tested [24]. The following open-ended questions were included:

1. Can you tell me more about the indigenous and traditional plants in your community/surroundings?

2. You have mentioned all these plants; let us talk about where you get them.

3. These plants, how do you use them?

4. These plants, how and why do you store them?

5. Please tell me more about the beliefs about these plants?

6. Please tell me more about your feelings and your views regarding these plants?

#### Concepts of knowledge and use

For the purpose of this study, ITPs and ALVs are foods/vegetables that are either native to the region, or were introduced to it a long time ago to evolve through natural processes or farmer selection, including both wild foods/vegetables and ones traditionally cultivated by the inhabitants of a region. In describing the results of the focus groups, it was important to contextualize the term “knowledge” and “use”. Gadgil *et al*. defined knowledge as an outcome of model-making about the functioning of the natural world. They further defined indigenous knowledge as “a cumulative body of knowledge and beliefs handed down through generations by cultural transmission about survival and the relationship of beings (including humans) with one another and their environment” [25]. This definition of knowledge was adapted to this study. In this context, parents were considered to be knowledgeable if they could express or give any form of relevant information related to the topic of study. The term “use” in this context referred to the purposes the ITPs served in the community and the processes that were involved in preparation to serve their purpose.

#### Data analysis

Household socio-demographic characteristics data was analysed by means of descriptive statistics using the IBM Statistical Package for Social Sciences (IBM SPSS 20.0 for Windows). A quality check was carried out on the transcribed data of the focus groups by a research assistant who was fluent in both English and Setswana, to ensure that the discussion was correctly translated without the original meaning being lost in translation. The notes taken during the focus group discussion by the assistant moderator were used to complement the rich data. The transcripts were coded and analysed with Atlas.ti 6 computer software, using the framework approach as described by Rabiee [26]. The coding of the transcripts was done independently by two researchers (MvdH and JO). After the second focus group no new codes were added. Differences between researchers were minimal and consensus was easily reached.

#### Trustworthiness

The principle of trustworthiness was adhered to using the approach of Guba (in [27]). The pilot study and the focus groups ensured prolonged engagement (truth value) with the participants in this study. Truth value was further increased through triangulation of investigators and sources. Applicability was ensured by conducting multiple focus groups, using a detailed interview guide, encouraging participants to share their knowledge, the saturation of data and a dense description of the methodology. Transferability was obtained through purposive sampling and using direct quotations when presenting findings. The establishment of an audit trial for stepwise replication of the research was possible and a co-coder was used during data analysis, ensuring consistency and neutrality.

### Phase 2: Child’s acceptance

#### Participants

Children from grade 2 to 4 in the two primary schools (n = 98; M/F: 40/58, 7–10 years) were randomly selected to participate in the sensory evaluation.

#### Food sample preparation and presentation

Four different dishes made from ALVs harvested in the study area and one dish made from store-bought Swiss chard were tested for acceptability. Swiss chard was included as reference sample. Each dish had a different vegetable content (see Table 1); however, the remaining ingredients were the same. These included tomatoes, onions, salt and vegetable oil. The ALVs selected for this study and the recipe for the dishes were based on the results of previous studies in the North West Province [28,29]. Sufficient samples of the five dishes were prepared, transported to the study site and heated in a microwave oven in a standardized manner. Each sample (numbered with three-digit random numbers) was served on a small, white plate accompanied by a small spoon.

**Table 1:** Leafy vegetables used in dishes tested in sensory evaluation

**Leafy vegetables used in dish**	**Common name**
ALV: *Amaranthus cruentus* L. (100%)	Amaranth
ALV: *Amaranthus cruentus* L. (80%) and *Cleome gynandra* L. (20%)	Amaranth and spiderplant
ALV: *Amaranthus cruentus* L. (80%) and *Cucurbita maxima* Lam. (20%)	Amaranth and pumpkin
ALV: *Amaranthus cruentus* L. (80%) and *Vigna unguiculata* (L.) Walp (20%)	Amaranth and cowpea
Conventional vegetable: *Beta vulgaris* L.	Swiss chard

#### Procedure for sensory evaluation

The sensory evaluation followed the procedures as described by Dalton *et al*. [30]. An hour before the sensory evaluation took place, the participant ate a sandwich with margarine and polony to prevent potential hunger from influencing the rating of the different dishes. The sensory evaluation took place in an empty classroom, in sessions of 10 participants per group. A facilitator conversed with the participants in the local language (Setswana) in a friendly manner to put them at ease. The facilitator explained the procedure and the score sheet to the group. Each participant was then allocated to a trained fieldworker for a one-on-one interview in the child’s preferred language. They were seated in such a way that interaction between the participants was minimised. Each child (n = 98) evaluated four of the five different samples (each dish was therefore evaluated at least 77 times), randomly allocated by means of a Latin square design. One by one, the participant evaluated each sample (30 gram) for colour, smell, taste and overall acceptance. Between tasting the samples the participants were ask to take a sip of water and eat a small piece of apple to cleanse the palate in order to reduce possible overlap of flavours. The participants could indicate their opinion by pointing at the relevant smiley face representing the score or whisper their response to the fieldworker. In both instances the fieldworker recorded the appropriate score. After the evaluation of four samples, the participant was asked if he or she had a preference for one of the samples and if so, for which sample. The last question was how many times per week the participant was willing to eat this type of food (leafy vegetables). The participants received a piece of candy as incentive after completion of the sensory evaluation.

#### Score sheet

**Figure 2:**
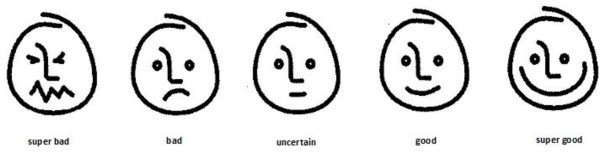
Five-point ordinal scale used for sensory evaluation (adapted from 30).

The procedure for sensory evaluation and the score sheet were standardised during a pilot study conducted in one of the schools. The 10 randomly selected children (M/F: 5/5; 7–10 years) participating in the pilot study were excluded from participation in the sensory evaluation. Chen *et al*. (1996) and Kroll (1990), both cited by Guinard [31], recommend using hedonic scales with verbal anchors. The score sheet used a five-point ordinal scale, ranging from super good (value = 5) to super bad (value = 1) (see Figure 2). The five-point ordinal scale has an equal number of positive and negative categories and is often used in consumer studies accommodating different language groups [32]. To avoid potential comprehension problems the five-point ordinal scale was explained by the facilitator using visual stimuli [31] in the form of a large lollipop (super good) and cod liver oil (super bad). The score sheet also included a question on a potential preferred sample and a seven-point food action rating scale to score consumption intent relating to each sample. This was done by asking how many times per week the participant was willing to eat leafy vegetables [29]. From the pilot study it was evident that the participants understood the score sheet used.

#### Data analysis

Each verbal anchor used in the sensory evaluation was allocated a value ranging from super good (value = 5) to super bad (value = 1). Mean values for the different attributes were calculated. Data was found not to be normally distributed, therefore the Kruskal-Wallis, Mann–Whitney U test and chi-square test for independence were performed using the IBM Statistical Package for Social Sciences (IBM SPSS 20.0 for Windows), controlling for Type 1 error across tests by using the Bonferroni approach. A p-value of <0.05 was regarded as significant.

## Results and discussion

### Parent’s knowledge, perceptions and use

In total, 29 parents (median age 40.1 years, range 20.7 – 82.9 years) participated in four focus groups. Most of them (n = 26) were female. This was expected, because women are usually responsible for taking care of children in the home and also are responsible for ensuring that the household has access to food. Table 2 describes the characteristics of these participants. More than half of the participants (58.6%) indicated that their husbands were the heads of the household, whereas 34% of the participants indicated that they themselves were the heads of the household. On average, two people (2.69 ± 1.58) in a household contributed to the income of the household. A child support grant was received by 69% of participants, whereas 19% received a pension grant. Most of participants indicated that their household did not receive any food from a feeding scheme (excluding the national school feeding programme) (89.7%) or that the household did not grow any food for its own use (72.4%). The socio-economic data indicated that these participants had a low educational background and were of low socio-economic status. This made them a vulnerable group, hence their significance for this study.

**Table 2:** Characteristics of focus group participants (n = 29)

	**n**	**Percentage**	**Median**	**Range**
**Education level**				
No education	5	17.2		
Primary education	14	48.3		
Secondary education	10	34.5		
Household size				
Children (0–18 years)			3.0	1-7
Adults (19–64 years)			2.0	0-5
Adults (65 and older)			0.0	0-2
Number of children taking care of			3.0	1-7
Work status				
Employed	17	58.6		
Unemployed/retired	12	41.4		
Total monthly household income				
Less than ZAR 1000	6	20.7		
ZAR 1000 – ZAR 2000	12	41.4		
ZAR 2000 – ZAR 3000	7	24.1		
More than ZAR 3000	4	13.8		
Monthly expenditures on food				
Less than ZAR 400	8	27.6		
ZAR 400 – ZAR 800	13	44.8		
More than ZAR 800	8	27.6		

ZAR: South African Rand; 1 USD ≈ 9.08 ZAR.

Direct quotations from participants were included in the description of the findings in order to give a richer description of the context. Results of the focus groups in general showed that the participants appeared to be knowledgeable on various edible ITPs in their surroundings in terms of their use.

### Edible ITPs

The themes that emerged from the data with regard to the edible ITPs included availability and access, preservation and storage, preparation and perceptions, including beliefs and feelings.

Table 3 shows the edible ITPs identified by the participants. The most commonly used edible plants were *Amaranthus spp*., commonly known as *thepe*, and *Chenopodium album*, commonly known as *senkgampapa*. The parts of the plants that were mostly consumed were the leaves. Participants referred to the leafy vegetables as “morogo” or “wild spinach”.

**Table 3:** Identified edible ITPs

**Local name in Setswana**	**Botanical name**	**Part consumed**	**FG 1**	**FG 2**	**FG 3**	**FG 4**
Amadumbe (IsiZulu)	*Colocasia esculenta* (L.) Schott and *Xanthosoma sagittifolium* (L.) Schott	Leaves, stem	-	Y	-	-
Bobete	*Urtica urens* L.	Leaves	Y	-	-	-
Chencha-keledi	Unknown	Fruit	-	Y	-	-
Kgobe-di-metsing	*Portulaca oleracea* L.	Leaves	-	-	-	Y
Leleme-la-kgomo	Looks like *Ricinodendron rautanenii* Schinz	Fruit, seeds	Y*	Y*	-	-
Lekatane	*Citrullus lanatus* (Thunb.) Matsum. & Nakai	Fruit, leaves	-	-	Y	-
Lekgomane	*Lagenaria siceraria* (Molina) Standl.	Leaves	Y	Y	-	-
Lerotho	*Cleome gynandra* L.	Leaves	Y	Y	-	-
Leshabe	*Sonchus asper* Vill. and *S. oleraceus* L.	Leaves	Y	-	-	-
Motangtang/Mistrikadika (fruit)	*Momordica balsamina* L.	Fruit	-	Y	-	-
Mmilo	*Vangueria infausta* Burch.	Fruit	-	Y	-	Y
Mmoko	Unknown	Fruit	-	Y	-	-
Moetsa-wa-pere	Unknown	Roots	Y*	-	Y	-
Mokofi	Unknown	Seeds	-	-	-	Y
Morwetla	*Grewia flava* D.C.	Fruit	-	Y	Y	Y
Monokotshwai	Unknown	Fruit	-	-	-	Y
Motswetswejane	Unknown	Fruit	-	-	Y	-
Qhela	Unknown	Leaves	-	-	-	Y
Rapa/rape	*Brassica rapa* L.	Leaves, stem	-	-	Y	-
Rotsane	Unknown	Fruit	-	Y	-	-
Sebitsa	Unknown	Leaves	-	-	Y	-
Sehuwe	Unknown	Leaves	Y	-	Y	Y *
Sekgalo	Unknown	Leaves	Y*	Y	Y	Y
Solele	*Portulaca oleracea* L.	Leaves	-	-	-	Y
Senkgane/Sekgapapane/ Imbikilicane	*Chenopodium album* L.	Leaves	Y*	Y*	Y*	Y*
Spaile	*Brassica carinata* A. Braun	Leaves	Y	Y*	-	Y*
Sepatlapatla	*Physalis peruviana* L.	Leaves, stem	Y	-	Y	-
Sthwanya	Unknown	Leaves	-	-	Y	Y
Storfyn	Unknown	Leaves	-	Y	-	-
Thepe	*Amaranthus spp*: *A. blitum* L., *A. graecizans* L., *A. cruentus* L., *A. tricolor* L.	Leaves, stem	Y*	Y*	Y*	Y *

The local names are given in Setswana, unless differently indicated. FG: Focus Group. (Y): the majority (75% or more) of participants within a particular focus group knew about the particular ITP mentioned and was familiar with its use. (*): the IPT was commonly/mostly used by the participants. (−): the ITP was not used and/or known.

Two of the identified plants, *Momordica balsamina* (Motangtang/Mistrikadika) and *Physalis pyruviana* (Sepatlapatla), were found to be used for both medicinal and food purposes. According to the participants, the fruits of the *Momordica balsamina* plant were eaten, mainly as a snack, whereas the leaves were used as eye medication for children. The leaves of the *Physalis pyruviana* plant were used as a condiment for starchy foods (mostly porridge made with maize meal, locally called “pap”). It was also known to cure ailments associated with pain.

Participants of focus group three and four were less familiar with some of the identified ITPs. A possible reason for this might be that these participants were mostly working or living closer to the urban area than those of the other two groups. When asked during the focus group discussions about the ITPs that they used, they tended to mention more exotic, cultivated plants such as cabbage, spinach and apples. It was thus evident that participants in group three and four used fewer of the plants that grow naturally and as a result were less exposed to them. Urbanisation has been associated with loss of knowledge of ITPs and their use, even by people who live in rural areas [1,33]. Vorster *et al*. reported that access to a market had a negative influence on the use of traditional vegetables [9]. People who did not have easy access to places where food is sold tended to rely more on those edible plants that grow naturally.

None of the participants had a negative perception of ITPs, which is in contrast to studies that found that especially members of the younger generation would label ITPs as inferior and as “poverty foods” [9,34]. The current study included mostly Tswana people, while Dweba and Mearns included predominantly Zulu people in their study [34] and Vorster *et al.* Pedi, Amaphondo-Xhosa, Tembu-Xhosa, Shangaan and Zulu people [9]. Cultural differences probably contribute to the difference in perceptions of ITPs reported across studies. Participants in the current study perceived ITPs as advantageous, since there are no monetary costs involved in obtaining these edible plants. According to the socio-demographic data, a typical household with about eight people only had ZAR 601-800 (≈USD 66–88) to spend on household food monthly, which was insufficient to sustain the whole household.

#### Availability and accessibility

According to the participants the edible, leafy ITPs and fruits were mostly found in the bushes, on farms and in fields and areas where water is widely available:

“*We get all the types of wild spinach from the farms and fields*, *like thepe and senkgane. On these farms*, *they grow maize and sunflowers and more. The wild spinach will grow in between these crops*.”

Participants said that the leafy ITPs, especially *thepe* and *spaile*, were available at certain times of the year and it was during those periods that the ITPs were mostly used or being preserved for when they were not available in the wild:

“*Thepe grows when it rains*, *but you eat it around August*.”

Participants who work on the farms may have access to these plants because they can pick them while working on the farms. The availability of these plants, however, can be compromised if they grow among cultivated crops as the farmers, who may have less knowledge about them, may see them as weeds and hence destroy them with herbicides. Vorster *et al*. reported that ITPs are still perceived as weeds by research and extension personnel who criticize farmers for not keeping them under control [9]. Farmers and research and extension personnel should thus be informed of the importance of these plants.

Participants indicated that the seasonal availability of most ITPs affected the frequency of consumption. In addition, drought periods affect their availability as well. Ways of improving the availability of ITPs during off-season and drought periods include using recycled water [9] and collection of seeds of ITPs. Collection of seeds is not the current practice in rural communities, where people rely on the plants’ self-sowing abilities of ITPs [35].

#### Preservation, storage and preparation

Participants were knowledgeable on how to access these plants during periods of low availability. One of the storage methods described was first to dry the leaves in the sun and then to store them in sacks. During storage, other plants (e.g. beans) were used as preservatives for the plants being stored.

Only one-third of the participants (34.5%) had access to a working fridge or freezer, meaning that the majority had to rely on traditional methods of preservation and storage. Sun drying of leaves is a cheap and convenient way of preserving these plants, especially in this population, but it may lead to a great loss of essential micronutrients such as vitamins A and C [36,37]. Some participants said the leaves of the plants could be cooked before being dried and stored. According to Mnkeni *et al*., all vegetables should be blanched in steam before drying, to deactivate the action of enzymes and also to prevent the loss of some nutrients [38]. Ndawula *et al*. observed that blanching reduced the loss of β-carotene in cowpea leaves [36].

When describing how they used the leafy ITPs, participants said that these vegetables were mostly used as condiments to accompany starch-based dishes (especially “pap”). Participants had different opinions on which ingredients to use during preparation of the leafy ITPs in order to enhance the taste.

“*With thepe you pick it and then cook it the way you know* … *if you have potatoes*, *you can add them. If you have onions*, *you can add them and also a bit of milk. You do not add water. If you have any spices*, *you add them*, *and there you will have a nice meal that you can enjoy with pap*.”

“*Then you add salt*, *maybe spices*, *and then eat it. It does not really need all that stuff*, *like*, *potatoes*, *tomatoes. To really enjoy thepe*, *you can just cook it and add onions and salt*.”

The leaves of the ITPs were often picked by hand, washed, and boiled in water. Certain types of leafy ITPs, such as spiderplant, were said to have a bitter taste, and as a result were boiled with plenty of water. Similar to the findings reported by Dweba and Mearns [34], this water was discarded two to three times in an attempt to get rid of the bitterness. Participants had mixed views and beliefs on the effect the rinsing and draining had on leafy ITPs. Some believed that the rinsing was good, as it improved the taste, while others did not think it was good because of nutrient losses:

“…*when you cook it*, *if you do not want to lose the nutrients*, *you do not pour out the water you cook it in*, *and you should not cook it for too long. You can add more vegetables to it* … *when you pour out the water that is when you lose all the vitamins. If you fry it until it is golden*, *you will lose all the nutrients and it will not be healthy for you* … *you must time it properly so that you do not overcook it and lose all the nutrients*.”

“*We wash it*, *put it in a pot*, *and boil it for maybe 30 minutes* … *my grandmother believed that you would drain out the vitamins*.” (The water was not drained.)

“*It tends to be very bitter*, *so you have to drain it* … *yes*, *it also has vitamins*.”

Draining and discarding the water used in cooking vegetables are likely to cause the loss of water-soluble vitamins such as vitamin B complex and vitamin C [34]. It is therefore important to educate this population on cooking methods that retain most of the nutritional value of the ITPs.

#### Perceptions, beliefs and feelings

When participants were asked about how they feel about the ITPs in their surroundings, the responses they gave were mostly positive and were related to the benefits these plants have for their wellbeing. The responses were related to the role the ITPs play in improving their health, the monetary benefits and lastly, the acceptability of these plants.

Edible ITPs were perceived as good sources of nutrients, especially vitamins, which they believed were essential in providing energy, boosting the immune system and preventing illnesses and infections. Similar to the findings of Nesamvuni *et al*. [10], participants were passionate and knowledgeable about these plants, especially wild spinach, and their importance in good nutrition. In contrast, Vorster *et al*. found that although ITPs were perceived to be nutritious because they had been consumed by previous generations, there is little awareness of the importance of these plants [9].

Participants felt that the edible plants were crucial in their lives because they always provided them with a source of food and they did not have to spend money to acquire these plants.

“*I feel proud of them because most of the time when you do not have money*, *you just go out*, *find them and collect them. Sometimes*, *you may have pap but nothing to eat it with. Then you can look for wild spinach*, *and you go to bed with a full stomach*.”

Throughout the focus group discussions participants expressed their acceptance of the taste of edible ITPs. The variety of ways in which they could be prepared with different ingredients made them very enjoyable to add as part of a meal. This is contrary to the findings of Dweba and Mearns, who reported that the lack of variety in cooking methods of traditional vegetables could make them less appealing and therefore affect consumption [34]. Participants also expressed a high preference for the leafy ITPs compared to meat.

Several studies have reported the nutritional composition of ALV, indicating that these vegetables are rich in various micronutrients such as β-carotene, iron, calcium, magnesium, zinc and vitamin C [6,7]. Increased availability of and access to these plants may thus help to address micronutrient deficiencies. ITPs could also play a role in the diversification of diets and improving household food security in resource-poor households [34,39]. Matenge *et al*. showed that consumption of these foods could be increased through education, increased availability, marketing and gradual introduction to ITPs [29]. This, combined with a positive attitude to ITPs, could benefit the promotion of ITPs. The concept of affordability should be avoided or used carefully in marketing strategies, as some ethnic groups/cultures associate these foods with poverty [7].

### Transfer of knowledge

Several methods for preservation and storage had been passed on through generations, thus indicating transfer of knowledge from one generation to another:

“*Back then*, *our mothers used to dry them and save them for the future*.”

In describing the cooking methods used for the various leafy ITPs, participants also indicated that there was transfer of knowledge through generations and hence the practice had not faded away completely:

“*I drain the water. Back home*, *my grandmother used to drain the water and then add peanuts and some oil. My grandmother believed they add*[*ed*] *flavour to the wild spinach and that peanuts were nutritious*.”

It is evident that the older generation in this population was seen as custodians of knowledge related to ITPs, and they passed their knowledge on in the hope that it would be transferred and not lost through generations. This finding was similar to the findings of a study conducted by Shava in the Eastern Cape, who found the younger generation to have extensive knowledge of ITPs in their surroundings, probably because of the close relationship they had with the elderly people in their community [40].

The focus groups were female-dominated, usually including only one male, and as a result females tended to dominate the discussions. However, the facilitator was able to insist on male participation regularly, and as a result, the males were able to describe their knowledge as well, thus avoiding bias. At times, some male participants were hesitant to make comments because they associated some aspects of the discussions with female roles, for example, cooking of food or the use of ITPs in the household. At times the older participants tended to hold back as the younger participants dominated the discussions. One elderly woman said: “*I just don*’*t understand* … *see the younger ones are managing well on their own with the information*”. The group facilitator was able to bridge these gaps and get the older participants more involved in the discussions as they went along. McLafferty stated that there are several ways of creating homogeneity in focus groups. In explorative studies homogeneity can also be classified according to status, class, occupation and other characteristics instead of gender and age [41]. The homogeneity between the participants in the focus groups was based on their role as parents of children and the person involved in food preparation, since the main focus of this study was on knowledge and usage of ITPs. For future studies, it may be more appropriate to separate the focus group participants by age and gender.

### Children’s sensory evaluation of African leafy vegetables

Although parents participating in the focus groups were knowledgeable about various edible ITPs and especially passionate about their importance in good nutrition, this study also sought to provide answers on children’s acceptance of ALVs by means of a sensory evaluation. Knowledge of children’s acceptance of the sensory attributes of ALVs is important for the potential future promotion of consumption of ITPs, including ALVs, as a strategy for reducing malnutrition (e.g. in a school nutrition programme).

The results of the sensory evaluation showed significant differences between the five dishes in the mean ratings for smell, taste and overall acceptability. When the dish made with Swiss chard was excluded from the analysis, there was no significant difference between any of the ratings of the four dishes made of ALVs (See Table 4).

**Table 4:** Evaluation of differences between the different dishes including and excluding the dish made with Swiss chard

	**Dishes including Swiss chard**		**Dishes excluding Swiss chard**	
	**K-W p-value**^**1**^	**P χ**^**2 **^**p-value**^**2**^	**K-W p-value**^**1**^	**P χ**^**2 **^**p-value**^**2**^
**Colour**	0.120	0.386	0.639	0.603
**Smell**	<0.001	0.003	0.426	0.261
**Taste**	<0.001	0.002	0.632	0.931
**Overall**	<0.001	0.039	0.599	0.931

p-value of <0.05 was considered statistically significant.

^1^Kruskal-Wallis p-value refers to mean values. ^2^Pearson χ^2^ p-value refers to frequency.

A comparison of the responses for gender revealed no statistically significant differences between different genders’ mean ratings for the five different dishes (Mann–Whitney U Test: p colour = 0.631; p smell = 0.268, p taste = 0.518 and p overall = 0.415). In the entire group, the dish made with Swiss chard was rated statistically significantly higher for smell, taste and overall acceptability than any of the dishes made with ALVs. There was no significant difference between the rating of the colour of Swiss chard and ALVs. Ratings for the dishes made with ALVs did not differ significantly in terms of colour, smell, taste and overall acceptability (see Table 5) and were thus equally acceptable regarding sensory characteristics to females and males. These four dishes combined were rated “good” or “super good” by 78.0%, 73.3%, 58.9% and 65.2% of the participants, respectively for colour, smell, taste and overall acceptability. Five of the 98 children did not have a preference for a specific dish. Of the 93 participants who did have a preference for one of the four dishes they evaluated, 75 had evaluated a dish made with Swiss chard. More than half (57.3%) of these participants preferred the sample made with Swiss chard. The median number of days per week the participants would like to eat these leafy vegetables was 2.0 (range 1–7). There was no statistically significant difference in the number of days per week the participants would like to eat these leafy vegetables between the participants who did and did not evaluate a dish made with Swiss chard (Mann–Whitney U test: p = 0.651). The intended consumption was consistent with the positive rating of the dishes made with ALVs.

**Table 5:** Sensory evaluation scores (mean ± SD) for different dishes made with leafy vegetables

**Dish**	**N**	**Colour**	**Smell**	**Taste**	**Overall**
100% Amaranth	77	3.83 ± 1.13^a^	3.66 ± 1.22^a^	3.53 ± 1.34^a^	3.82 ± 1.44^a^
80% Amaranth + 20% cowpea	80	4.05 ± 0.98^a^	3.95 ± 1.02^a^	3.50 ± 1.45^a^	3.71 ± 1.35^a^
80% Amaranth + 20% pumpkin	79	3.85 ± 1.16^a^	3.65 ± 1.25^a^	3.33 ± 1.47^a^	3.57 ± 1.47^a^
80% Amaranth + 20% spiderplant	78	3.83 ± 1.19^a^	3.85 ± 1.17^a^	3.31 ± 1.41^a^	3.59 ± 1.48^a^
100% Swiss chard	78	4.21 ± 0.93^a^	4.33 ± 0.94^b^	4.26 ± 1.19^b^	4.38 ± 1.13^b^

Score: five-point ordinal scale ranging from 5 (super good) to 1 (super bad). Dishes with different superscript differed statistically significant (p < 0.05).

The dish made with Swiss chard was included as a reference dish, as it was expected that children would be exposed to this vegetable more often at home and at school via the daily cooked school meal. This was evident in the preference for the dish made with Swiss chard with regard to smell and taste. Although the dish made with Swiss chard was preferred, the four dishes made with ALVs were found to be of acceptable colour, smell and taste. These results are promising for the inclusion of ALVs in children’s diet, particularly as Michicich *et al*. found a positive correlation between liking and consumption [42]. Therefore, ALVs might be successfully introduced into a school nutrition programme that has a limited budget per school meal. Developing a range of recipes with different ingredients, as mentioned in the focus group discussions, will add variety to the flavours of the dishes. ALVs have been advocated as excellent sources of several micronutrients [6,7] and could potentially address co-existing multiple micronutrient deficiencies in individuals. Edible ITPs can add variety to the diets of people, especially those who do not have easy and regular access to markets or other fresh produce.

## Conclusion

This study indicated that there was not only a wealth of knowledge on various edible ITPs and their use in this farm community, but traditionally prepared ALV dishes were also sensorily acceptable to children. Edible ITPs were usually found growing on farms and fields among cultivated crops, and their availability and accessibility were influenced by seasonality and environmental conditions. Drying and storage methods for use during off-season periods should be optimized to minimize nutrient losses. Edible ITPs were perceived as rich sources of health-promoting nutrients and an affordable source of food and were appreciated for their taste. Knowledge about edible ITPs was transferred from one generation to another. The positive perceptions and knowledge of ITPs of the parents, and the children’s acceptance of the taste of dishes made with ALVs indicate great potential for the promotion of ITPs as a strategy for improved child nutrition. It also looks promising for future use of edible ITPs in, for example, school feeding programmes. The researchers will be investigating the effect of these ALVs as part of the school meal on the nutritional status of children. Results of this study to date strongly indicate that compliance in the intervention study will be high.

## Abbreviations

ALVs: African leafy vegetables; ITPs: Indigenous and traditional plants.

## Competing interests

The authors declare that they have no competing interests.

## Authors’ contributions

MvdH: design; acquisition of data; analysis and interpretation of data; drafting the manuscript. JO: acquisition of data; analysis and interpretation of data; drafting the manuscript. MG: design; critical revision of manuscript for intellectual content. AK: critical revision of manuscript for intellectual content. MF: design; critical revision of manuscript for intellectual content. CMS: design; critical revision of manuscript for intellectual content. All authors read and approved the final manuscript.

## Authors’ information

MvdH is currently a PhD student in Nutrition at the Centre of Excellence for Nutrition (CEN) at the North-West University, South Africa. She is studying the effect of African leafy vegetables on the alleviation of micronutrient deficiencies in school children residing in the North West Province of South Africa. She is also the research co-ordinator for the South African leg of the Biodiversity study at Africa Unit for Transdisciplinary Health Research (AUTHeR) at the same university. This transdisciplinary research aims to increase agricultural biodiversity to improve nutritional and health status, as well as livelihoods and to establish more sustainable production systems in the North West Province of South Africa.

JO is currently a PhD student in Nutrition at the CEN at the North-West University, South Africa. Her PhD research is focused on iodine nutrition in mothers and their infants in the North West Province, South Africa. She has just completed her MSc in Nutrition; her research focused on the potential contribution of African leafy vegetables to the nutritional status of school children residing in the North West Province of South Africa.

MG is a professor and senior researcher in the AUTHeR in the Faculty of Health Sciences of the North-West University, South Africa. She holds an MCur and a PhD in Psychiatric Nursing. She is an acknowledged researcher and has published extensively in national and international scientific journals and presented her research findings at many national and international conferences. Her research over the past few years has mainly focused on the quality of life of people living with HIV and AIDS and HIV stigma reduction on a community base. She is a South African nationally rated researcher and an inducted member of the International Nurses Hall of Fame.

MF is a chief specialist scientist at the Medical Research Council and an extra-ordinary professor at the University of the Western Cape as well as the University of Pretoria, South Africa. She is an acknowledged researcher and her research over the past few years has mainly focused on food-based approaches to address micronutrient malnutrition in children, particularly in rural areas. Her research also covers aspects of food security and indigenous foods.

CMS is a professor in nutrition and senior scientist in the CEN, North-West University, South Africa. He is currently the president of the Nutrition Society of South Africa. He is a well-recognised researcher and has an interest in the role of essential fatty acids in health and disease, and the interactions between iron and essential fatty acids in cognitive development. His research mostly targets vulnerable children residing in low socio-economic areas.
